# GSI Treatment Preserves Protein Synthesis in C2C12 Myotubes

**DOI:** 10.3390/cells10071786

**Published:** 2021-07-15

**Authors:** Joshua R. Huot, Brian Thompson, Charlotte McMullen, Joseph S. Marino, Susan T. Arthur

**Affiliations:** 1 Laboratory of Systems Physiology, Department of Kinesiology, University of North Carolina at Charlotte, Charlotte, NC 28223, USA; jrhuot@iu.edu (J.R.H.); brianthompson072@gmail.com (B.T.); charlottemc27@gmail.com (C.M.); joseph.marino@uncc.edu (J.S.M.); 2 Department of Surgery, Indiana University School of Medicine, Indianapolis, IN 46202, USA

**Keywords:** muscle protein synthesis, GSI, mTOR, AKT

## Abstract

It has been demonstrated that inhibiting Notch signaling through γ-secretase inhibitor (GSI) treatment increases myogenesis, AKT/mTOR signaling, and muscle protein synthesis (MPS) in C2C12 myotubes. The purpose of this study was to determine if GSI-mediated effects on myogenesis and MPS are dependent on AKT/mTOR signaling. C2C12 cells were assessed for indices of myotube formation, anabolic signaling, and MPS following GSI treatment in combination with rapamycin and API-1, inhibitors of mTOR and AKT, respectively. GSI treatment increased several indices of myotube fusion and MPS in C2C12 myotubes. GSI-mediated effects on myotube formation and fusion were completely negated by treatment with rapamycin and API-1. Meanwhile, GSI treatment was able to rescue MPS in C2C12 myotubes exposed to rapamycin or rapamycin combined with API-1. Examination of protein expression revealed that GSI treatment was able to rescue pGSK3β Ser9 despite AKT inhibition by API-1. These findings demonstrate that GSI treatment is able to rescue MPS independent of AKT/mTOR signaling, possibly via GSK3β modulation.

## 1. Introduction

Skeletal muscle wasting is a debilitating result of aging and several disease states, which drastically reduces functional capacity and quality of life [1,2,3]. Loss of skeletal muscle mass can be attributed to increased protein catabolism, impaired muscle regeneration (i.e., myogenesis), and/or reductions in muscle protein synthesis (MPS) [1,4,5]. The protein kinase B (AKT)/mechanistic target of rapamycin (mTOR) cascade is pivotal for several processes within skeletal muscle including survival, autophagy, differentiation, and MPS [6,7]. Interestingly, emerging evidence has identified mTOR as a primary antagonist of lifespan, revealing that administration of rapamycin (a potent inhibitor of mTOR) can increase lifespan, improve aging, and combat age-related disease development [8,9,10,11,12,13]. However, reduced mTOR signaling in skeletal muscle diminishes myogenic potential, and reduces anabolic potential of exercise and nutrients [14,15]. This poses an interesting dilemma for skeletal muscle researchers, in particular when seeking to maintain muscle mass in diseased and aged populations.

Another pathway strongly implicated in skeletal muscle health and disease is Notch signaling [1]. Notch is activated when one of its four receptors (Notch1–4) binds to one of several Notch ligands (Delta-like protein (DLL)1, DLL3, DLL4, Jagged1, Jagged2), which initiates successive metalloprotease and γ-secretase cleavages [1]. Aside from its developmental regulation, Notch signaling dictates the myogenic response following injury [16,17]. Similar to mTOR, dysfunctional Notch signaling may also occur in atrophic skeletal muscle, blunting skeletal muscle regeneration [16,18,19,20]. Moreover, aberrant Notch signaling has been implicated in the development of insulin resistance and cachexia [21,22,23]. Meanwhile, targeting the γ-secretase cleavage via γ-secretase inhibitors (GSIs), a commonly used method to chemically inhibit Notch signaling, has demonstrated that reduced Notch signaling increases myotube formation and muscle growth [22,24,25]. Altogether, these findings make Notch an interesting target to potentially combat muscle atrophy in aging and other skeletal muscle wasting diseases.

Reducing mTOR activity can promote healthy aging, yet at the same time blunts the anabolic potential of skeletal muscle. Thus, identifying and targeting signaling pathways that modulate MPS independent of mTOR may help to sustain skeletal muscle mass in the aging population. One example of this is AKT, which can mediate MPS via glycogen synthase kinase 3 beta (GSK3β) independently of mTOR [26,27,28]. Interestingly, our lab recently demonstrated that Notch inhibition via GSI treatment elevated MPS in C2C12 myoblasts and myotubes by modulating the phosphatase and tensin homolog (PTEN)/AKT/mTOR signaling cascade [25]. However, it is not known if this mechanism is reliant on mTOR or if GSIs mediate MPS in an AKT-dependent manner. 

Thus, in the present study we sought to investigate whether the beneficial effects of GSIs on myotube size and MPS were dependent on AKT/mTOR. Here, we again demonstrate that GSI treatment increases differentiation and MPS in C2C12 myotubes. Inhibition of AKT and mTOR ablated GSI-induced differentiation in C2C12 cells. However, GSI treatment preserved MPS rates in combination with AKT and mTOR inhibition, suggesting that the use of GSIs may be able to augment MPS independent of AKT/mTOR. 

## 2. Materials and Methods

### 2.1. Cell Culture

For all in vitro experiments, C2C12 skeletal muscle myoblasts (ATCC p3–p8) were cultured in Dulbecco’s Modified Eagles Medium (DMEM), supplemented with 10% fetal bovine serum, 10% horse serum (HS), and 1% penicillin/streptomycin (P/S), as performed previously [25]. In particular, we previously demonstrated that γ-secretase inhibitor (GSI-4 µM: L-685,458; Millipore Sigma- dimethyl sulfoxide (DMSO)) treatment on the onset of C2C12 differentiation increases myotube fusion and AKT/mTOR signaling [25]. Thus, to examine if GSI-mediated effects on myotube fusion were dependent on AKT/mTOR, we exposed C2C12s at the onset of differentiation to 4 µM GSI in combination with 100 nM rapamycin (RAP; 13346; Cayman Chemicals in DMSO) or 10 µM 4-Amino-5,8-dihydro-5-oxo-8-b-D-ribofuranosyl-pyrido[2,3-d]pyrimidine-6-carboxamide (API-1; SML1342; Millipore Sigma in DMSO), established inhibitors of mTOR and AKT, respectively [29,30,31]. Specifically, for the differentiating C2C12 experiments, myoblasts were grown to full confluence, switched to differentiation media (DM: DMEM supplemented with 2% HS and 1% P/S) and treated every 12 h for 96 h in one of the following conditions: (1) GSI, RAP, GSI + RAP, or control (Con: DMSO). (2) GSI, API-1, GSI + API-1, or Con. Myotubes from these experiments were analyzed for indices of fusion and area as outlined below. In addition to demonstrating that GSI treatment enhances C2C12 differentiation, we also previously reported that differentiated myotubes exposed to GSI for 24 h was sufficient to increase MPS [25]. Thus, to determine if the GSI-mediated effects on MPS in differentiated myotubes were reliant on AKT/mTOR signaling, C2C12s were allowed to differentiate for 72 h and were then treated twice (every 12 h) for the next 24 h under one of the following conditions: (1) GSI, RAP, GSI + RAP, or Con; (2) GSI, API-1, GSI + API-1, or Con; (3) GSI, RAP + API-1, GSI + RAP + API-1, or Con. Myotubes from these experiments were analyzed for protein expression, protein synthesis, and myotube diameter, as detailed below.

### 2.2. Myosin Heavy Chain Staining

Following 96 h of differentiation, myotubes were stained with myosin heavy chain (MHC) and assessed for properties of fusion, area, and diameter, as performed previously [25,32]. Briefly, myotubes were fixed with 70% acetone/30% methanol, serially washed with PBS, blocked for 1 h, and incubated overnight in MHC (MF-20, 1:100; Developmental Studies Hybridoma Bank, Iowa City, IA, USA). Myotubes were subsequently PBS-washed, incubated with an anti-mouse secondary antibody (1:200) and 4′,6-Diamidino-2-Phenylindole, Dihydrochloride (DAPI 1:1000) for 1 h, and mounted with Vectashield.

### 2.3. Myotube Fusion, Area, and Diameter

Stained myotubes were captured at 20× for indices of fusion and area on an Olympus iX inverted microscope, as performed previously [25]. Following image acquisition, 2 blinded individuals quantified indices of myotube fusion, including myotube number, total nuclei, and fused nuclei using ImageJ. Myotube area was determined from the same images used to calculate fusion index using Adobe Photoshop, as previously described [33]. Briefly, three randomly selected images from each experimental group (Con, GSI, RAP, GSI + RAP or Con, GSI, API-1, GSI + API-1) were used to set accepted tones for MHC (red) and DAPI (blue). The set color range was then subsequently applied to all images in order to obtain measures for total myotube area, area per myotube, and myotube area per fused nuclei. For myotube size, ImageJ software was used to measure the narrowest diameter along the myotube (400 myotubes per condition) [32,34]. 

### 2.4. Protein Synthesis

For assessment of protein synthesis, myotubes were treated with 1 µM puromycin (P-1033, A.G. Scientific, San Diego, CA, USA) 30 min prior to cell collection, as previously described [25,35,36]. Puromycin incorporation was subsequently analyzed via western blot, as detailed below.

### 2.5. Western Blot

To extract protein from C2C12 myotubes, well surfaces were washed two times with cold PBS, mechanically lysed in chilled Radioimmunoprecipitation assay (RIPA) buffer (sc-24948; Santa Cruz Biotechnology, Dallas, TX, USA) containing 1% Triton-x, 2% SDS and protease cocktail inhibitors, as performed previously [25], and centrifuged for 20 min at 20,000× *g* (4 °C). Following centrifugation (20,000× *g*, 20 min, 4 °C), the supernatant was saved and assessed for protein concentration by the BCA protein assay method (23225; ThermoFisher, Waltham, MA, USA). Samples (20 µg) were loaded and electrophoresed on a 4–12% Bis-Tris gel (3450125; Bio-Rad, Hercules, CA, USA) at 125 V for 2 h, as performed previously [25]. Proteins were then transferred (Towbin Buffer; 10% methanol) onto a 0.22 µM Polyvinylidene difluoride (PVDF) membrane for 1 h at 100 V. Membranes were washed in Tris-buffered saline (TBS), blocked for 1 h in Odyssey blocking buffer (1:1 TBS), and incubated overnight in primary antibodies. Following primary antibody incubation, membranes were serially washed in TBST (TBS: 0.1% Tween 20) and incubated in secondary antibodies (1:10,000 in TBST) for 1 h. Membranes were again serially washed in TBST, and proteins were then visualized and quantified using the Odyssey^®^ Licor CLx System. Antibodies used were as follows: pAKT Thr308 (#13038; 1:500), pAKT Ser473 (#4060; 1:500), AKT (#2920; 1:1000), pmTOR Ser2448 (#5536; 1:500), mTOR (#4517, 1:1000), p4EBP1 Thr37/46 (#2855; 1:500), 4EBP1 (#9644; 1:1000), pp70S6K Thr389 (#9234; 1:500), p70S6K (#2708; 1:1000), peEF2 Thr56 (#2331; 1:500), eEF2 (#2332; 1:1000), pGSK3β Ser9 (#8566; 1:500), GSK3β (#5676; 1:1000), and ABC (#8814; 1:1000) from Cell Signaling; Puromycin (#MABE343; 1:5000) from EMD Millipore; and β-Actin (#A2228; 1:10,000) from Sigma Aldrich, St. Louis, MI, USA.

### 2.6. Statistical Analysis

One-way analysis of variance (ANOVA) tests were performed to determine differences between experimental groups (1) GSI, RAP, GSI + RAP, or Con. 2) GSI, API-1, GSI + API-1, or Con. 3) GSI, RAP + API-1, GSI + RAP + API-1, or Con). Post-hoc comparisons were accomplished via a Tukey’s test, with statistical significance set apriori at *p* ≤ 0.05. All statistical analyses and graphs were made using Graphpad Prism 7.03 (GraphPad, San Diego, CA, USA). All data are presented as means ± SD.

## 3. Results

### 3.1. Rapamycin Ablates GSI-Mediated Elevations in Myotube Formation

**Figure 1:**
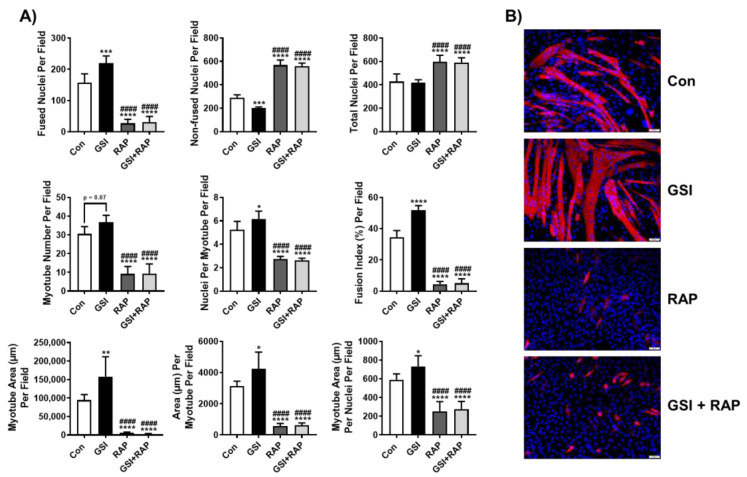
Rapamycin ablates GSI-mediated elevations in myotube formation. (**A**) Indices of myotube fusion and area. Graph order, top left to right: Fused nuclei per field, Non-fused nuclei per field, and Total nuclei per field. Graph order, middle left to right: Myotube number per field, Nuclei per myotube per field, Fusion index per field. Graph order, bottom left to right: Myotube area (µm) per field, Area (µm) per myotube per field, Myotube area (µm) per nuclei per field. (**B**) Representative image of 96-h myotubes co-stained with myosin heavy chain (MHC:red) and DAPI:blue. Images were taken at 20× magnification and the scale bar = 50 µm. At the onset of differentiation C2C12 cells were treated every 12 h with either control (Con), 4 µM γ-secretase inhibitor (GSI), 100 nM rapamycin (RAP), or GSI + RAP co-treatment. All data were analyzed using a one-way ANOVA followed by Tukey’s multiple comparison test. * *p* < 0.05, ** *p* < 0.01, *** *p* < 0.001, **** *p* < 0.0001 vs. Con; #### *p* < 0.0001 vs. GSI (*n* = 3 experiments). Data are mean ± SD.

Since our work previously demonstrated that Notch inhibition via GSI treatment was sufficient to increase myotube formation as well as mTOR signaling, we wanted to determine whether GSI-mediated effects on myogenesis were dependent on mTOR [25]. Thus, we treated differentiating C2C12 myotubes with GSI and the commonly used mTOR inhibitor, rapamycin, for 96 h. Similar to our previous reported results, GSI treatment significantly increased several indices of myotube formation compared to all groups, including fused nuclei per field, nuclei per myotube per field, and fusion index per field (Figure 1). GSI treatment also resulted in significantly reduced non-fused nuclei compared to all other groups (Figure 1). Interestingly, RAP significantly reduced fused nuclei, nuclei per myotube, and fusion index compared to Con, but did not differ from GSI + RAP in any of these measures, suggesting that GSI-mediated increases in fusion are dependent on mTOR (Figure 1). Moreover, both RAP and GSI + RAP had significantly elevated non-fused nuclei compared to Con, while also increasing total nuclei per field compared to Con and GSI, yet did not differ from each other (Figure 1). The myotube number was also significantly reduced in RAP and GSI + RAP compared to both Con and GSI (Figure 1). With respect to area measures, GSI treatment increased total myotube area, area per myotube, and myotube area per fused nuclei (Figure 1). Meanwhile, RAP and GSI + RAP both reduced myotube area, area per myotube, and myotube area per fused nuclei compared to Con, further suggesting that GSI-mediated increases in myotube formation are dependent on mTOR (Figure 1).

### 3.2. GSI Treatment Preserves Protein Synthesis in the Presence of Rapamycin 

**Figure 2:**
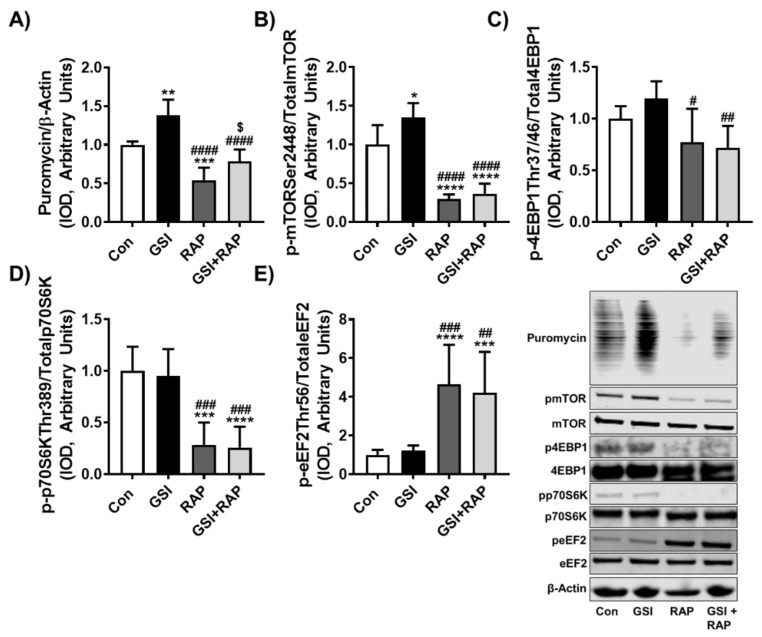
GSI treatment rescues protein synthesis in the presence of rapamycin. Representative western blotting and quantification (expressed as fold change vs. control (Con) for (**A**) Puromycin/β-Actin (**B**) Phospho (p)-mTOR Ser2448/Total mTOR; (**C**) p-4EBP1 Thr37/46/Total 4EBP1; (**D**) p70S6K Thr389/Total p70S6K; and (**E**) p-eEF2 Thr56/Total eEF2. Next, 72 h post-differentiation, C2C12 cells were treated every 12 h with either Con, 4 µM γ-secretase inhibitor (GSI), 100 nM rapamycin (RAP), or GSI + RAP co-treatment until 96 h post-differentiation. Then, 30 min prior to collection, all cells were treated with 1 µM puromycin. All data were analyzed using a one-way ANOVA followed by Tukey’s multiple comparison test. * *p* < 0.05, ** *p* < 0.01, *** *p* < 0.001, **** *p* < 0.0001 vs. Con; # *p* < 0.05, ## *p* < 0.01, ### *p* < 0.001, #### *p* < 0.0001 vs. GSI; $ *p* < 0.05 vs. RAP (*n* = 3 experiments). Data are mean ± SD.

We previously demonstrated that GSI treatment increased MPS in differentiating and differentiated C2C12 myotubes [25]. As the present data demonstrated that the use of rapamycin ablated GSI-augmented fusion, we wanted to determine whether the effects of GSI on MPS were also reliant on mTOR. To do this we differentiated C2C12 myotubes for 72 h and then exposed them to GSI and RAP for 24 h. Confirming our published work, GSI treatment increased protein synthesis compared to all other groups, while RAP exhibited reduced protein synthesis compared to Con (Figure 2A). Interestingly, GSI treatment protected protein synthesis rates and myotube size in the presence of RAP (Figure 2A and Figure S1). In addition, in line with our prior work, GSI treatment increased phosphorylated (p)-mTOR at Ser2448 compared to all other groups, while p-mTOR was reduced compared to Con in both RAP and GSI + RAP treated myotubes, suggesting that GSI preservation of MPS is not dependent on mTOR (Figure 2B). Moreover, despite increasing p-mTOR, GSI treatment did not exert effects directly downstream of mTOR (p-4EBP1, p-p70S6K, p-eEF2), which is comparable to our prior findings (Figure 2C–E) [25]. Meanwhile, RAP and GSI + RAP did not differ in any downstream target of mTOR, showing reduced 4EBP1, reduced p-p70S6K, and increased p-eEF2 (Figure 2B–D). These results suggest that GSI treatment may protect MPS levels in the presence of rapamycin and thus may modulate MPS in mechanisms other than mTOR.

### 3.3. API-1 Ablates GSI-Mediated Elevations in Myotube Formation

**Figure 3:**
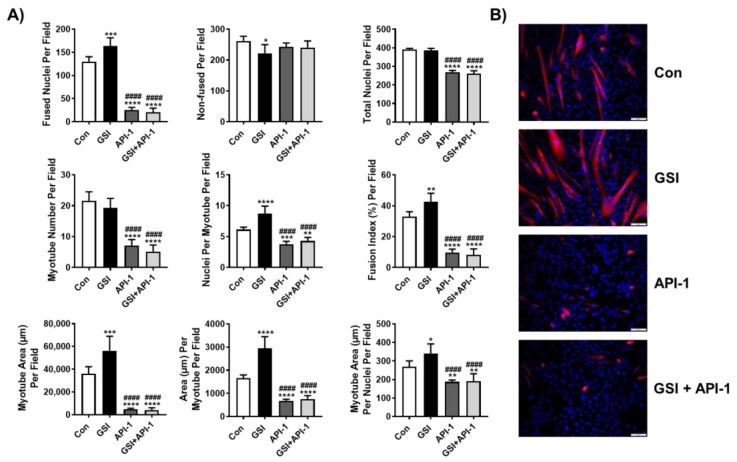
API-1 ablates GSI-mediated elevations in myotube formation. (**A**) Indices of myotube fusion and area. Graph order, top left to right: Fused nuclei per field, Non-fused nuclei per field, and Total nuclei per field. Graph order, middle left to right: Myotube number per field, Nuclei per myotube per field, Fusion index per field. Graph order, bottom left to right: Myotube area (µm) per field, Area (µm) per myotube per field, Myotube area (µm) per nuclei per field. (**B**) Representative image of 96-h myotubes co-stained with myosin heavy chain (MHC:red) and DAPI:blue. Images were taken at 20× magnification and the scale bar = 50 µm. At the onset of differentiation C2C12 cells were treated every 12 h with either control (Con), 4 µM γ-secretase inhibitor (GSI), 10 µM 4-Amino-5,8-dihydro-5-oxo-8-β-D-ribofuranosyl-pyrido[2,3-d]pyrimidine-6-carboxamide (API-1), or GSI + API-1 co-treatment. All data were analyzed using a one-way ANOVA followed by Tukey’s multiple comparison test. * *p* < 0.05, ** *p* < 0.01, *** *p* < 0.001, **** *p* < 0.0001 vs. Con; #### *p* < 0.0001 vs. GSI (*n* = 3 experiments). Data are mean ± SD.

To expand upon our prior findings that GSI treatment promotes fusion as well as increased AKT signaling in C2C12s, we also decided to investigate if AKT was necessary for GSI-mediated effects on myotube formation [25]. To do this we treated differentiating C2C12s with GSI and an AKT inhibitor, API-1. In concert with previous experiments, GSI treatment increased all measured markers of myotube formation and fusion. GSI treatment increased fused nuclei, nuclei per myotube, and fusion index compared to all other groups (Figure 3). API-1 and GSI + API-1 treatment induced lower fused nuclei, nuclei per myotube, and fusion index compared to Con, but did not differ from each other in any parameter (Figure 3). GSI treatment also reduced non-fused nuclei compared to Con; however, there were no differences between the other groups (Con; API-1; GSI + API-1) (Figure 3). The lack of difference in non-fused nuclei between groups is likely due to the significant reduction seen in total nuclei with API-1 and GSI + API-1 treatment (Figure 3). Similar to reductions in total nuclei per field, the myotube number was also reduced in API-1 and GSI + API-1 compared to Con and GSI (Figure 3). Regarding area measures, total myotube area, area per myotube, and myotube area per fused nuclei were increased with GSI treatment compared to all groups (Figure 3). Further suggesting that API-1 treatment ablates myotube formation induced by GSI treatment, API-1 and GSI + API-1 had reduced total myotube area, area per myotube, and myotube area per fused nuclei compared to Con, but did not differ from each other in any parameter (Figure 3). 

### 3.4. GSI Treatment Preserves Protein Synthesis in the Presence of API-1 and Rapamycin

**Figure 4:**
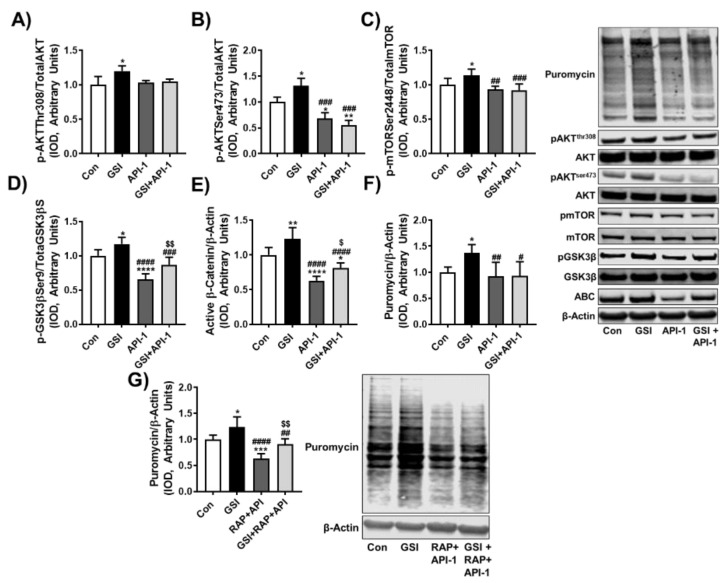
GSI treatment rescues protein synthesis in the presence of API-1 and rapamycin. Representative western blotting and quantification (expressed as fold change vs. control (Con) for (**A**) Puromycin/β-Actin; (**B**) Phospho (p)-AKT Thr308/Total AKT; (**C**) p-AKT Ser473/Total AKT; (**D**) p-mTOR Ser2448/Total mTOR; (**E**) p-GSK3β Ser9/Total GSK3β; (**F**) Active β-Catenin/β-Actin. Then, 72 h post-differentiation, C2C12 cells were treated every 12 h with either Con, 4 µM γ-secretase inhibitor (GSI), 10 µM 4-Amino-5,8-dihydro-5-oxo-8-β-D-ribofuranosyl-pyrido[2,3-d]pyrimidine-6-carboxamide (API-1), or GSI + API-1 co-treatment until 96 h post-differentiation. All cells were treated with 1 µM puromycin 30 min prior to collection. Representative western blotting and quantification for (**G**) Puromycin/β-Actin. Next, 72 h post-differentiation, C2C12 cells were treated every 12 h with either Con, 4 µM GSI, 100 nM rapamycin (RAP) + 10 µM API-1, or GSI + RAP + API-1 co-treatment until 96 h post-differentiation. All cells were treated with 1 µM puromycin 30 min prior to collection. All data were analyzed using a one-way ANOVA followed by Tukey’s multiple comparison test. * *p* < 0.05, ** *p* < 0.01, *** *p* < 0.001, **** *p* < 0.0001 vs. Con; # *p* < 0.05, ## *p* < 0.01, ### *p* < 0.001, #### *p* < 0.0001 vs. GSI; $ *p* < 0.05, $$ *p* < 0.01 vs. API-1 (**D**,**E**); $$ *p* < 0.01 vs. RAP + API-1 (**G**) (*n* = 3 experiments). Data are mean ± SD.

Given our prior findings that GSI-treated myotubes have increased AKT signaling along with increased MPS and our present findings that GSI treatment protected MPS in the presence of rapamycin, we wanted to determine whether GSI-mediated effects on MPS are dependent on AKT (Figure 2) [25]. This was also of interest as AKT can mediate MPS independently of mTOR by way of GSK3β [26,27,28]. In line with our prior findings, GSI treatment increased MPS and myotube size compared to all groups; however, the use of API-1 was not sufficient to reduce MPS compared to Con (Figure 4A and Figure S2). In addition, MPS with GSI + API-1 was no different than API-1 alone, suggesting that GSI-mediated effects on MPS may be dependent on AKT (Figure 4A). However, despite not reducing MPS, API-1 was sufficient to reduce myotube size, while introduction of GSI in the presence of API-1 preserved the myotube size (Figure S2). Interestingly, though GSI treatment increased phosphorylation of AKT on both Thr308 and Ser473, the use of API-1 was only sufficient to reduce pAKT Ser473 (Figure 4B,C), and GSI + API-1 did not differ from API-1 at either phosphorylation site (Figure 4B,C). Downstream of AKT, GSI treatment significantly elevated pmTORSer2448 compared to all groups (Figure 4D). Intriguingly, and similar to pAKT Thr308, API-1 and GSI + API-1 did not reduce pmTORSer2448 compared to Con, nor were they different from each other, suggesting that GSI treatments elevation of mTOR may be dependent on AKT. In addition, the fact that pmTOR did not reduce with API-1 treatment may also explain the lack of reduction in MPS.

Since AKT also mediates protein synthesis independently of mTOR by phosphorylating GSK3β, we wanted to assess if this signaling was changed with GSI and with API-1 treatments. GSI-treated C2C12s demonstrated elevations in pGSK3βSer9 (Figure 4E), and while API-1 did not cause reductions in mTOR, it did reduce pGSK3βSer9 compared to Con (Figure 4E). Intriguingly, pGSK3βSer9 was elevated in GSI + API-1 compared to API-1 and did not differ from Con. Moreover, GSI treatment increased active β-catenin (ABC) compared to all groups and GSI + API-1 was sufficient to preserve ABC compared to API-1 alone (Figure 4F). The preservation of pGSK3βSer9 with GSI treatment in combination with API-1 convinced us to observe protein synthesis conditions in which MPS would surely be reduced. Thus, we conducted an additional experiment set utilizing GSI + RAP + API-1. Again, GSI treatment increased MPS compared to all other groups (Figure 4G). Treatment of C2C12s with RAP + API-1 significantly reduced MPS and myotube size compared to Con, while the introduction of GSI in the presence of RAP + API-1 preserved both MPS and myotube size (Figure 4G and Figure S3).

## 4. Discussion

Our lab recently demonstrated that Notch inhibition via GSI treatment elevates protein synthesis in C2C12 muscle cells, possibly through modulation of the AKT/mTOR signaling cascade [25]. In the present study, we expand upon our prior findings and demonstrate that GSI treatment may be able to augment MPS independent of AKT/mTOR, as GSI treatment in the presence of mTOR and AKT inhibition was able to provide protection of protein synthesis in C2C12 myotubes.

One goal of the present study was to determine if the GSI-mediated elevations in myotube formation and growth was dependent on mTOR, as mTOR is a pivotal regulator of myogenesis [37,38,39]. Here, we show that GSI treatment significantly elevates a myriad of fusion and hypertrophy indices (fusion index, area/myotube, and myotube area/fused nuclei) in differentiating C2C12 myotubes and that these elevations are completely ablated by the introduction of rapamycin. This is in full support of the notion that mTOR is required for muscle cell differentiation and demonstrates that GSI-mediated myogenesis enhancement is dependent on mTOR. In contrast, we demonstrated that GSI treatment protects MPS and myotube size in differentiated C2C12s exposed to rapamycin, suggesting that GSI may modulate MPS in an mTOR-independent manner.

Given AKT’s ability to regulate myotube differentiation and elevate protein synthesis independent of mTOR through regulation of GSK3β, myotubes were also exposed to the AKT inhibitor, API-1 [26,40,41]. In a similar fashion, API-1 diminished GSI-mediated enhancements on C2C12 differentiation, which is in concert with prior findings that AKT is essential for the initiation of myoblast differentiation [41]. However, API-1 alone was not sufficient to reduce mTOR or MPS in differentiated myotubes. API-1 is a novel small molecule inhibitor that has gained research attention in recent years as a possible anti-cancer therapeutic, and, while evidence shows reduced pAKTSer473, to our knowledge it is unclear if both pAKTThr308 and Ser473 are reduced with API-1 treatment [31,42,43,44]. Here, we show that API-1 only reduced pAKTSer473 and not pAKTThr308. This may explain why we did not observe detectable reductions in mTOR signaling or MPS with API-1 treatment. Though it is reported that reductions in AKT can blunt protein synthesis, other proteins may influence mTOR independent of AKT, which could explain why the use of API-1 is not sufficient enough to reduce protein synthesis [6,7,45]. In contrast, API-1 treatment did reduce another downstream target of AKT, pGSK3βSer9 and subsequent ABC protein expression, demonstrating that the use of API-1 does negatively influence the AKT function. Furthermore, despite its lack of effect on MPS, API-1 treatment did reduce myotube size. AKT is a known pro-survival signaling pathway and API-1 has shown to induce apoptosis, thus the atrophy observed in the present study is likely due to heighted protein breakdown and not a suppression of MPS [46,47,48]. However, we did not measure specific indices of protein catabolism in the present study and can only speculate at this time.

An underlying premise of this study was to gain better insight into the mechanisms by with GSI treatment elevates MPS in C2C12 myotubes, and since API-1 alone was not sufficient to reduce protein synthesis, myotubes were exposed to API-1 and RAP together. Intriguingly, GSI treatment was able to rescue protein synthesis rate and myotube size in the presence of both API-1 and RAP, suggesting that GSI may mediate MPS in a mechanism independent of AKT/mTOR. Our data suggests that this rescue of MPS may be via GSK3β. When active, GSK3β phosphorylates and inactivates the translation initiation factor eIF2B [28,40]. However, when GSK3β is phosphorylated on Ser9 by AKT, eIF2B is able to partake in translation initiation [49]. As mentioned above, pGSK3βSer9 was significantly reduced with API-1 treatment. Interestingly, pGSK3βSer9 was rescued with GSI treatment in the presence of API-1, suggesting that GSIs may act on GSK3β independent of AKT. In concert with this finding, ABC was also rescued, indicating that function of GSK3β may be modulated by GSIs independent of AKT. This is not the first time that GSIs or Notch and GSK3β crosstalk have been postulated. In fact, during investigations using HEK293T cells, smooth muscle cells and fibroblasts have identified Notch as a target of GSK3β [50,51,52]. Interestingly, however, work within skeletal muscle has also suggested a regulatory role of Notch on GSK3β. Brack et al. discussed GSK3β as a mediator between Notch and Wnt during skeletal muscle regeneration, demonstrating GSI-mediated elevations in pGSK3βSer9 similar to our present findings [16]. However, their work did not discuss GSK3β as a mediator of protein synthesis and did not investigate whether GSI treatment impacts MPS. GSK3β is a known regulator of MPS and overexpression of its downstream target eIF2Bε has shown to significantly increase rates of MPS and induce muscle hypertrophy [40,53]. Additionally, Notch signaling has been implicated in regulating muscle hypertrophy in both in vivo and in vitro models [22,54,55,56]. To our knowledge, we are the first to address the research paradigm of Notch as a regulator of MPS and have shown for the first time that GSI treatment may modulate MPS independently of mTOR/AKT. An interesting yet puzzling finding from our lab’s prior work was that 4EBP1 was the only downstream target of mTOR that was altered in a protein synthesis positive fashion with GSI treatment. This may actually support the idea that GSI-mediated elevations or rescue of MPS is through GSK3β. In fact, recent studies within cancer cell lines have implicated GSK3β directly in the phosphorylation of 4EBP1 [57,58]. It is also interesting that GSK3β has been implicated in regulating PTEN stabilization [59,60]. Based on our previous published work demonstrating alterations in the PTEN/AKT/mTOR cascade, it is plausible that GSI treatment’s modulation of PTEN/AKT/mTOR is via GSK3β.

Though our present study reveals that GSIs may be able to rescue MPS when combined with mTOR and AKT inhibitors, this study is not without limitations. Though we have validated GSI treatment as a strategy to increase MPS in vitro, whether GSIs augment MPS in vivo requires further investigation. It should be noted that GSI administration did counteract muscle wasting in a setting of cachexia, but in this case MPS was not assessed [22]. Additionally, though GSIs are routinely used to target Notch signaling, they are not specific to Notch, and have several additional target substrates [61,62]. A large majority of γ-secretase substrates are transmembrane receptors, much like the Notch receptor family, and have known roles within skeletal muscle. For example, the insulin receptor, insulin-like growth factor 1 receptor, and growth hormone receptor, all of which partake in anabolic signaling, have been identified as γ-secretase substrates [62]. However, to our knowledge, the regulation of these receptors by GSIs have not been investigated specifically within skeletal muscle. Another γ-secretase substrate, low-density lipoprotein receptor-related protein 6 (LRP6), is a transmembrane receptor crucial to canonical Wnt signaling [62,63]. Crosstalk between Notch and Wnt have been widely discussed for proper skeletal muscle regeneration, while LRP6-mTOR signaling has been investigated in cardiomyocytes and hepatocytes [1,64,65]. Yet, whether LRP6-mTOR signaling occurs in skeletal muscle or whether LRP6 is responsible for changes in MPS is unknown. Recent work has also demonstrated that inhibition of the receptor for advanced glycation end products (RAGE), a γ-secretase substrate, can partially protect against age-associated muscle atrophy [66]. Further, a proteomics approach following GSI treatment in chick myogenic cells identified sonic hedgehog (Shh) as one of the most altered signaling pathways. Interestingly, separate studies have shown that Shh promotes myoblast proliferation, while also increasing myoblast fusion [67,68]. Yet, to our knowledge, the role of Shh on MPS has not been investigated. Thus, given the vast array of γ-secretase substrates, future investigations must delineate whether Notch is the specific GSI target that is responsible for alterations in MPS reported in the present study. Lastly, though we have demonstrated that administration of a GSI is able to promote pGSK3βSer9 in the presence of API-1, and thus are speculating that GSI-mediated rescue of MPS is via GSK3β, future work will be required to confirm this mechanism of action.

## 5. Conclusions

This study provided additional validation for the use of GSIs to promote or rescue MPS. We demonstrated that GSI treatment can rescue MPS independently of AKT and mTOR, possibly through regulation of GSK3β. These findings warrant further investigation on the role of GSIs in muscle-wasting conditions, in particular instances in which tumor-suppressing drugs (Rapamycin, API-1) are used, or where MPS rates are reduced, as GSIs may elevate MPS and help to sustain skeletal muscle mass.

## Data Availability

Data sharing is not applicable to this article.

## Supplementary Materials

The following are available online at https://www.mdpi.com/article/10.3390/cells10071786/s1, Figure S1: GSI preserves myotube size in the presence of rapamycin, Figure S2: GSI preserves myotube size in the presence of API-1, Figure S3: GSI preserves myotube size in the presence of rapamycin and API-1.
