# Supplementary material for: GSI Treatment Preserves Protein Synthesis in C2C12 Myotubes
Source: Cells. 2021 Jul 15;10(7):1786. doi: 10.3390/cells10071786 (PMC8307118; doi:10.3390/cells10071786)
Supplement: Supplementary file 1 [file cells-10-01786-s001.zip › cells-1257396-supplementary.pdf]

Supplementary

GSI Treatment Preserves Protein Synthesis in C2C12 Myotubes

Joshua R. Huot, Brian Thompson, Charlotte McMullen, Joseph S. Marino and Susan T. Arthur

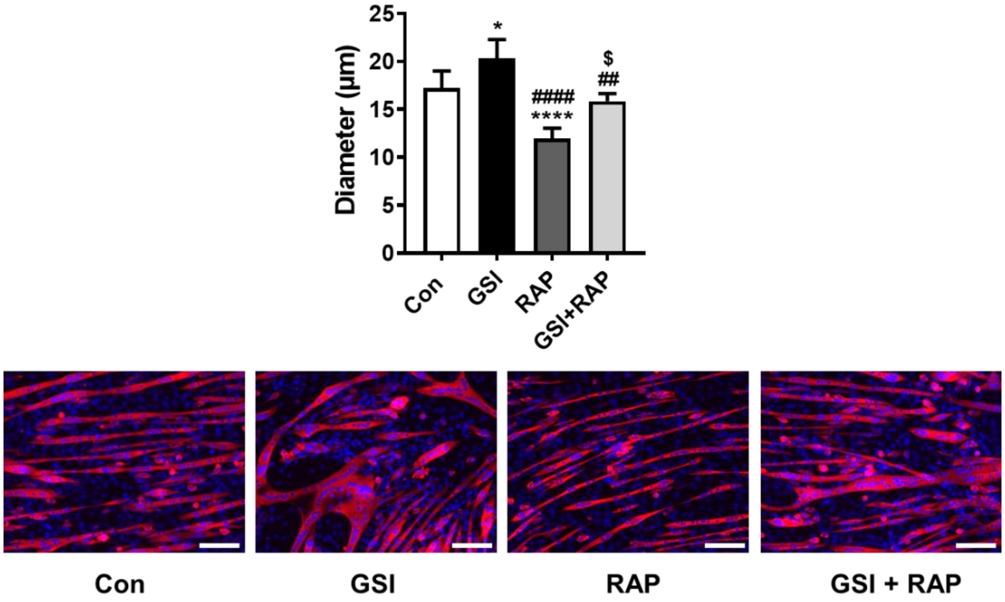

Figure S1. GSI preserves myotube size in the presence of rapamycin. Quantification of myotube diameter (400 per condition) and representative images of 96-hour myotubes co-stained with myosin heavy chain (MHC) and DAPI. Images were taken at 10x magnification and the scale bar = 100 µm. 72 hours post-differentiation, C2C12 cells were treated every 12 hours with either Con, 4µM γ-secretase inhibitor (GSI), 100nM rapamycin (RAP), or GSI + RAP co-treatment until 96 hours post-differentiation. All data were analyzed using a one-way ANOVA followed by Tukey's multiple comparison test. *$p < 0.05$, ****$p < 0.0001$ vs. Con; ##$p < 0.01$, ####$p < 0.0001$ vs. GSI; \$$p < 0.05$ vs. RAP. Data are mean ± SD.

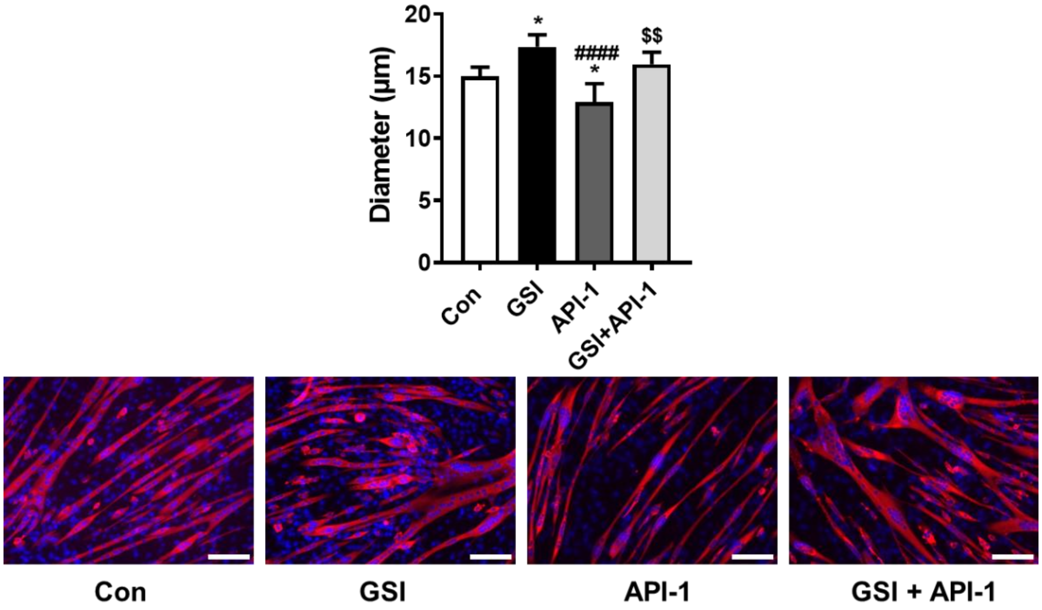

Figure S2. GSI preserves myotube size in the presence of API-1. Quantification of myotube diameter (400 per condition) and representative images of 96-hour myotubes co-stained with myosin heavy chain (MHC) and DAPI. Images were taken at 10x magnification and the scale bar = 100 µm. 72 hours post-differentiation, C2C12 cells were treated every 12 hours with either Con, 4µM γ-secretase inhibitor (GSI), 10µM 4-Amino-5,8-dihydro-5-oxo-8-β-D-ribofuranosyl-pyrido[2,3-d]pyrimidine-6-carboxamide (API-1), or GSI + API-1 co-treatment until 96 hours post-differentiation. All data were analyzed using a one-way ANOVA followed by Tukey's multiple comparison test. *$p < 0.05$, vs. Con; ####$p < 0.0001$ vs. GSI; $$p < 0.01 vs. API-1. Data are mean ± SD.

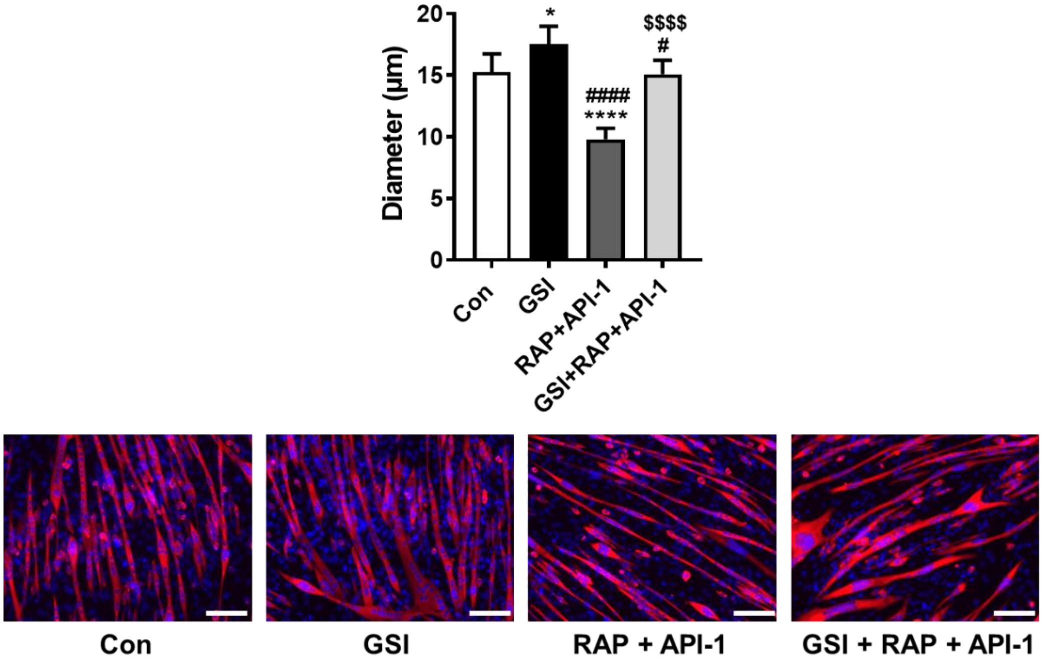

Figure S3. GSI preserves myotube size in the presence of rapamycin and API-1. Quantification of myotube diameter (400 per condition) and representative images of 96-hour myotubes co-stained with myosin heavy chain (MHC) and DAPI. Images were taken at 10x magnification and the scale bar = 100 µm. 72 hours post-differentiation, C2C12 cells were treated every 12 hours with either Con, 4µM γ-secretase inhibitor (GSI), 100nm rapamycin (RAP) + 10µM 4-Amino-5,8-dihydro-5-oxo-8-β-D-ribofuranosyl-pyrido[2,3-d]pyrimidine-6-carboxamide (API-1), or GSI + RAP + API-1 until 96 hours post-differentiation. All data were analyzed using a one-way ANOVA followed by Tukey's multiple comparison test. *$p < 0.05$, ****$p < 0.0001$ vs. Con; #$p < 0.05$, ####$p < 0.0001$ vs. GSI; $$$$$p < 0.0001$ vs. RAP+API-1. Data are mean ± SD.
